# A pan‐genome framework for exploiting germplasm diversity and structural variation in rose breeding

**DOI:** 10.1002/imo2.70125

**Published:** 2026-07-14

**Authors:** Abdelhafid Bendahmane

**Affiliations:** ^1^ Institute of Plant Sciences Paris‐Saclay (IPS2) Université Paris‐Saclay, CNRS, INRAE, Université d'Evry Gif‐sur‐Yvette France

## Abstract

The urgent need for diverse rose cultivars requires better use of germplasm resources. Zhang et al. generated a rose pan‐genome from 26 accessions, revealing structural variation and introgression linked to flowering, double flowers, and petal color, providing a valuable resource for molecular rose breeding.

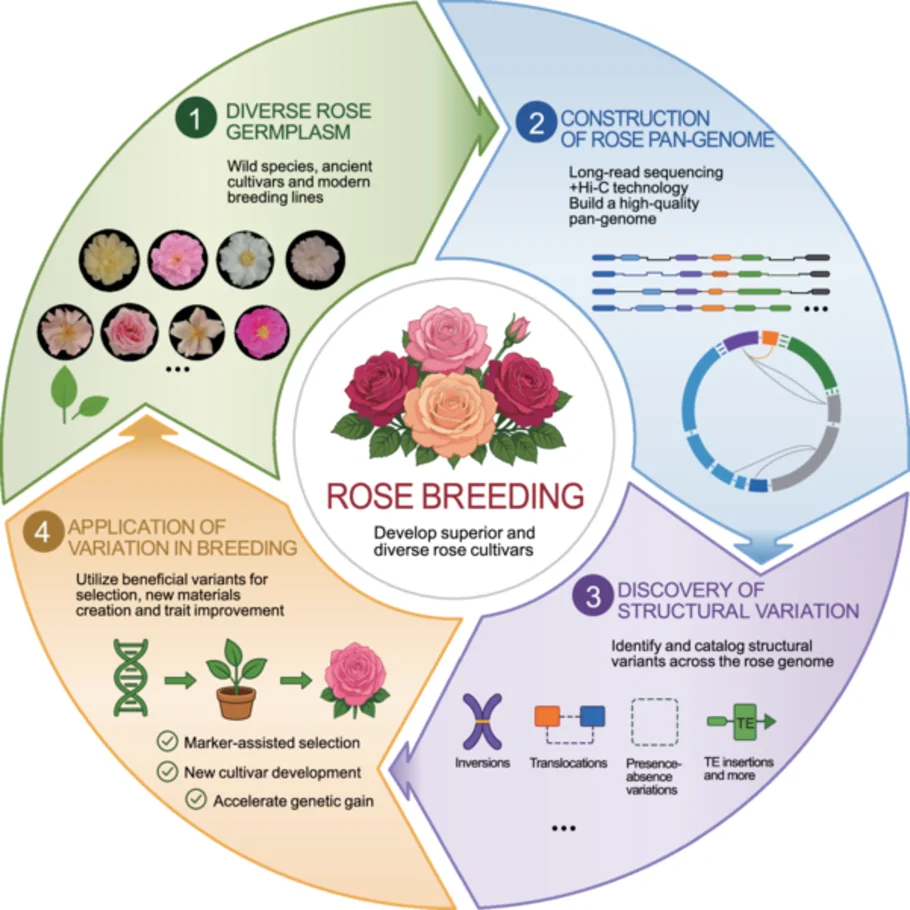

Roses (*Rosa* spp.) are among the most economically important ornamental crops worldwide, valued for their remarkable diversity in flowering behavior, floral morphology, and petal color. These traits underpin their global horticultural and cultural significance and have been shaped by extensive interspecific hybridization and long‐term breeding [1]. Previous genomic and evolutionary studies have shown that repeated hybridization events among wild and cultivated roses have played a central role in generating phenotypic diversity [2, 3]. However, the genetic basis of this diversity remains incompletely resolved, largely due to the complex genomic architecture of roses, characterized by high heterozygosity and frequent polyploidization within the subgenus *Rosa*.

Although hybridization contributes to diversity, it does not fully explain how genomic variation is translated into stable phenotypic traits. Recent advances in plant genomics highlight the importance of structural variation, including inversions, presence–absence variation and transposable element insertions, in shaping gene regulation and trait evolution in complex genomes [4, 5, 6]. However, how these sources of variation can be systematically captured and exploited in roses remains unclear. In a recent study, Zhang et al. constructed a haplotype‐resolved pan‐genome of the subgenus *Rosa*, providing a comprehensive framework to address this challenge [7].

## 1 PAN‐GENOME AS A COMPREHENSIVE ROSE GERMPLASM RESOURCE

Recent genomic resources have substantially advanced rose research, including the development of high‐quality reference genomes, population genomic datasets, and trait‐mapping studies [2, 8]. These efforts have facilitated the identification of loci underlying important ornamental traits such as flowering behavior, floral morphology, fragrance, and petal color. However, despite increasing knowledge of rose evolutionary history, a comprehensive understanding of how genome‐wide structural variation contributes to trait diversification across diverse germplasm has remained lacking. The haplotype‐resolved pan‐genome developed by Zhang et al. provides a powerful framework to address this challenge [7].

Consistent with this, Zhang et al. identified widespread introgression across rose lineages based on phylogenetic analyses, highlighting the pervasive impact of hybridization on the genomic landscape of *Rosa* [7]. Similar patterns of reticulate evolution have also been reported in other Rosaceae species, further supporting hybridization as a general mechanism shaping ornamental plant diversity. The study further resolved the hybrid origin and subgenome composition of the allotetraploid *Rosa gallica*, as well as the genetic background of the first modern rose cultivar “La France” (Figure 1A). These findings refine previous evolutionary models and confirm that hybridization provides the genetic foundation for phenotypic diversification. However, this process alone does not fully explain how specific traits emerge or stabilize.

**Figure 1:**
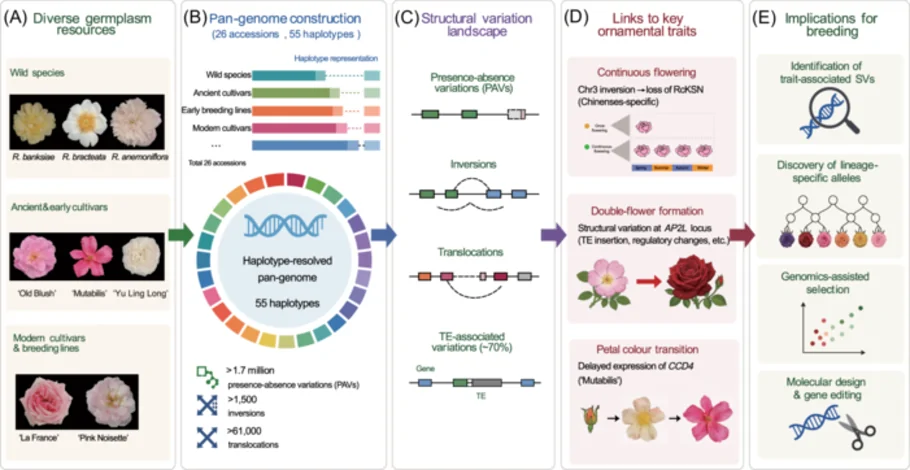
Pan‐genome‐guided framework for rose germplasm utilization and trait improvement. A haplotype‐resolved pan‐genome constructed from diverse *Rosa* germplasm reveals extensive structural variation and links genome architecture to key ornamental traits, providing a basis for genomics‐assisted rose breeding. (A) Diversity of wild and cultivated rose germplasm. (B) Haplotype‐resolved pan‐genome construction in *Rosa*. (C) Genome‐wide landscape of structural variation. (D) Structural variation underlying ornamental trait diversification. (E) Implications for rose genomics and molecular breeding.

## 2 STRUCTURAL VARIATIONS AS A MAJOR SOURCE OF TRAIT DIVERSITY

In plant genomics, increasing evidence indicates that structural variation contributes substantially to genome evolution, often exceeding the functional impact of single‐nucleotide polymorphisms in highly heterozygous species. This is particularly relevant in perennial and polyploid plants, where genome restructuring is frequent [9, 10, 11].

In the pan‐genome constructed by Zhang et al., extensive structural variation was identified across wild species, ancient cultivars, and modern breeding lines. Using long‐read and Hi‐C sequencing, the authors generated highly contiguous assemblies and detected over 1.7 million presence–absence variations, more than 1500 inversions and over 61,000 translocations (Figure 1B,C). Similar magnitudes of structural variation have been reported in other complex plant genomes, such as potato [4] and wheat [12], where TE‐driven genome dynamics play a central role in diversification. In roses, a large proportion of these variants are associated with transposable elements, suggesting that TE activity is a major force driving genome restructuring. These structural changes influence gene function through disruption, dosage variation, and regulatory rewiring, providing a functional layer of diversity beyond sequence polymorphism.

## 3 STRUCTURAL VARIATION LINKS GENOME CHANGE TO ORNAMENTAL TRAITS

Deciphering the genetic basis of ornamental traits requires connecting genome‐scale variation to phenotypic outcomes. Previous studies in plants have shown that structural variation can directly affect key agronomic traits by altering gene dosage [13], disrupting coding regions [5], or modifying regulatory landscapes [14]. Recent studies have also identified key genetic variants underlying floral scent diversity in roses, highlighting the contribution of natural variation to aroma‐related traits [15, 16].

Zhang et al. identified structural variants affecting previously characterized key regulatory genes such as *RcKSN* and *AP2L*, providing new insights into the allelic and structural basis of well‐known flowering and floral morphology regulators across different rose lineages [7]. For example, a Chinensis‐specific inversion on chromosome 3 is associated with loss of *RcKSN* function and the continuous‐flowering phenotype, a defining trait in modern roses. At the *AP2L* locus, multiple independent structural mechanisms contribute to double‐flower formation across diverse germplasm. In addition, delayed expression of *CCD4* underlies the dynamic petal color transition in the cultivar ‘Mutabilis’, affecting carotenoid degradation and producing gradual color shifts during development (Figure 1D). Together, these results demonstrate that structural variation plays a central role in converting genomic diversity into phenotypic innovation.

## 4 IMPLICATIONS FOR ROSE EVOLUTION AND BREEDING

Beyond its conceptual insights into rose evolution and trait diversification, the haplotype‐resolved pan‐genome constitutes an important genomic resource for the broader research community. Previous genomic studies have largely relied on individual reference genomes, which are unable to fully capture the extensive diversity generated by hybridization, heterozygosity, and polyploidization within *Rosa*. By establishing a haplotype‐resolved pan‐genome spanning diverse related wild species and cultivated roses, Zhang et al. provide a comprehensive framework for investigating genome evolution and trait diversification at the genus level [7]. The resulting resource enables systematic analyses of structural variation, facilitates the discovery of lineage‐specific alleles, and offers new opportunities to connect evolutionary processes with phenotypic innovation. More broadly, this work demonstrates how pan‐genomic approaches can uncover the genetic basis of complex traits in highly heterozygous ornamental crops, providing a valuable model for future studies in roses and other Rosaceae species.

This study establishes a conceptual framework in which diverse germplasm provides the genetic reservoir, while structural variation reshapes genome architecture and gene function to generate phenotypic innovation (Figure 1E). This perspective extends previous models that primarily emphasized hybridization and highlights structural variation as a critical component in explaining trait diversity in roses.

From a breeding perspective, structural variants associated with desirable traits represent promising targets for genomics‐assisted improvement, while pan‐genomic resources provide a powerful platform for identifying lineage‐specific alleles across diverse germplasm. Evaluating the stability and transferability of trait‐associated variants across diverse germplasm and cultivation conditions will therefore be critical for their effective deployment in breeding programs. However, the functional effects of many structural variants remain to be validated, and their phenotypic impact may vary across genetic backgrounds and environments. Future integration of pan‐genomics with functional genomics and genome editing will be essential to fully exploit rose germplasm and accelerate molecular breeding.

## AUTHOR CONTRIBUTIONS

**Abdelhafid Bendahmane**: Writing—review and editing; manuscript approval.

## CONFLICT OF INTEREST STATEMENT

The author declares no conflicts of interest.

## ETHICS STATEMENT

No animals or humans were involved in this study.

## AI STATEMENT

ChatGPT (5.5) was used for grammar checking. All content has been verified for accuracy by author.

## Data Availability

Data sharing not applicable to this article as no datasets were generated or analysed during the current study. Supplementary materials (graphical abstract, slides, videos, Chinese translated version and update materials) may be found in the online DOI or iMeta Science http://www.imeta.science/imetaomics.
